# Neural Reactivity to Social Reward Moderates the Association Between Social Media Use and Momentary Positive Affect in Adolescents

**DOI:** 10.1007/s42761-024-00237-1

**Published:** 2024-05-22

**Authors:** Madison Politte-Corn, Samantha Pegg, Lindsay Dickey, Autumn Kujawa

**Affiliations:** 1 Department of Psychology, The Pennsylvania State University, 140 Moore Building, University Park, PA USA; 2 Department of Psychology and Human Development, Vanderbilt University, Nashville, TN USA

**Keywords:** Social media, Adolescent, Positive affect, Negative affect, EEG, Reward

## Abstract

Peer relationships take on increasing importance during adolescence, and there has been considerable debate about the effects of social media use on adolescent emotional health. Little work has examined individual differences in brain function that might impact these associations. In this study, we examined the reward positivity (RewP) to social and monetary reward as a moderator of the relation between social media use and concurrent momentary affect in adolescents. Participants were 145 adolescents aged 14–17 (*M* = 15.23; *SD* = 1.08; 64.1% female; 71.7% White) at varying risk for depression (47 high-risk based on maternal depression history, 50 low-risk, 48 currently depressed). Measures of social media use, positive affect (PA), and negative affect (NA) were obtained through ecological momentary assessment. In a laboratory session, adolescents completed a computerized peer feedback task and a monetary reward task to elicit the RewP to social and monetary reward feedback. Multilevel models indicated that social media use and a smaller RewP to monetary rewards were associated with lower PA. However, social (but not monetary) reward responsiveness moderated the effect of social media use on momentary PA, such that social media use was associated with lower PA for adolescents with a relatively blunted RewP to peer acceptance, but not for those with an enhanced social RewP. Exploratory analyses indicated that this moderation effect was specific to female adolescents. The results highlight neural reactivity to social reward as a potential factor contributing to variability in the effect of social media use on affective health.

Social media use is now a ubiquitous part of adolescents’ lives, with recent population-level data indicating that 62% of youth aged 13–18 spend more than two hours per day on social media on average (Rideout et al., 2022). Most research has focused on the association between social media use and internalizing symptoms in adolescents (Ivie et al., 2020; Kreski et al., 2021; Twenge et al., 2018), but social media use may have stronger effects on short-term markers of emotional functioning such as positive affect (PA) and negative affect (NA; Dienlin & Johannes, 2020; Politte-Corn et al., 2024). Moreover, adolescence is characterized by normative increases in neural sensitivity to social rewards such as peer acceptance (Nelson et al., 2016), and developmental neuroscience theories posit that this increased reactivity to peer feedback may interact with adolescents’ social environment to influence psychosocial outcomes (Blakemore & Mills, 2014; Schriber & Guyer, 2016). Given the key role that social rewards (e.g., the number of followers, likes, and positive comments) play in maintaining social media use (Lindström et al., 2021), individual differences in neurophysiological responses to social rewards likely shape the association between adolescent social media use and emotional health, yet this remains to be empirically tested.

Many studies have examined the relation between social media use and depressive symptoms (e.g., Bottaro & Faraci, 2022; Kreski et al., 2021; Twenge et al., 2018), with meta-analytic data indicating a significant but small positive association in adolescents (*r* = 0.11; Ivie et al., 2020). Mixed findings and small effects across this literature indicate the need for other approaches to examining the link between adolescent social media use and mental health outcomes. In particular, social media use may have stronger effects on more proximal markers of affective health, such as PA and NA, than more stable or distal outcomes, such as depressive symptoms, given that PA and NA are state-level measures that can be measured concurrently with social media use and are subject to greater within-person fluctuations (Diener et al., 2018; Dienlin & Johannes, 2020; Yasuda et al., 2004).

Consistent with this, prior work indicates a significant association between social media use and both PA and NA, though the direction of these effects varies across studies (Apaolaza et al., 2014; Drach et al., 2021; Hamilton et al., 2021; Politte-Corn et al., 2024). For instance, one study found that information-seeking, entertainment, and socializing on social media were associated with higher PA (Apaolaza et al., 2014), and other work indicates increases in PA following a negative mood induction when browsing social media compared to a control website (Drach et al., 2021). However, these studies measured trait-level affect, and ecological momentary assessment (EMA) studies examining state-level associations suggest an inverse relation between social media use and PA. Specifically, one EMA study demonstrated that compared to in-person interactions, positive and negative peer interactions on social media were more likely to be associated with reduced PA and higher NA (Hamilton et al., 2021). In our previous work using EMA with this sample, we found evidence that social media use was associated with lower levels of PA concurrently and at the next assessment, and lower NA at the next assessment for depressed youth (Politte-Corn et al., 2024). However, we also found considerable variability in the effect of social media use on NA (ranging from β = -0.47 to β= 0.21), with one-third of participants showing a positive association (Politte-Corn et al., 2024). This variability between and within studies could be due in part to individual differences that moderate this association, in line with the differential susceptibility to media effects model (Piotrowski & Valkenburg, 2015).

Importantly, the social brain undergoes both structural and functional changes during adolescence, coinciding with a shift from familial to peer interactions as the dominant social context (Blakemore, 2012; Nelson et al., 2016). Consequently, adolescence is characterized by heightened sensitivity to peer approval compared to earlier or later in development, which facilitates important developmental tasks such as peer integration (Blakemore & Mills, 2014; Foulkes & Blakemore, 2016; Nelson et al., 2016). At the same time, this heightened sensitivity to peer feedback is thought to interact with changes in adolescents’ social environment to influence psychosocial outcomes (Blakemore & Mills, 2014; Schriber & Guyer, 2016). The adolescent neurobiological sensitivity to context theory (Schriber & Guyer, 2016) posits that these brain-based individual differences make some adolescents more susceptible to both positive and negative developmental outcomes, depending on the social context with which they interact. Drawing from these frameworks, social media use may interact with neural sensitivity to peer feedback to make some adolescents more susceptible to both the harms and benefits of social media use.

Social media is characterized by numerous quantifiable metrics of peer acceptance (i.e., number of followers, likes, and comments), and emerging neuroscience models posit that social media use activates areas of the brain implicated in social reward processing, including the ventromedial prefrontal cortex (vmPFC) and ventral striatum (Doheny & Lighthall, 2023; Meshi et al., 2015). Moreover, Meshi et al. (2015) propose that some forms of online interactions may actually activate social reward regions, particularly the ventral striatum, to a greater degree than in-person interactions. Although the relation between social media use and neural social reward responsiveness has not yet been empirically examined, there is some neurobiological and behavioral evidence that supports this link. Specifically, a recent study demonstrated that adolescents who showed heightened activation in the vmPFC, a brain region involved in reward processing and valuation (Lin et al., 2012; Moretti et al., 2009), while viewing the faces of popular peers reported higher PA on days when they used social media more (Maza et al., 2024). Moreover, adolescents with higher vmPFC activation while viewing low popular peers reported higher NA on days when they used social media more, suggesting that neural valuation of popularity shapes the relation between daily social media use and affect (Maza et al., 2024). Behaviorally, computational modeling indicates that social media engagement conforms to a reward learning paradigm in which the receipt of social rewards (in the form of likes) predicts future posting behaviors (Lindström et al., 2021). Further, an EMA study with adolescents found that PA while using social media was related to within-person increases in social media checking behaviors, providing additional behavioral evidence that social media use is rewarding (Dreier et al., 2023) and raising questions about how individual differences in reward systems function might shape responses to social media.

Despite the potential rewards that come with social media use, there appears to be heterogeneity in its immediate impacts on PA. Specifically, some studies report associations between social media use and higher PA (Apaolaza et al., 2014; Drach et al., 2021), whereas others indicate that lower PA is associated with social media use (Hamilton et al., 2021; Politte-Corn et al., 2024). This could be due in part to individual differences that moderate the effects of social media use on affect. In particular, trait-like aspects of reward systems functions could be relevant. These individual differences in reward processing can be reliably characterized at the neurophysiological level via the reward positivity (RewP), an event-related potential derived from EEG which indexes reactivity to both monetary and social reward feedback (Foti et al., 2011; Proudfit, 2015). Due to the low spatial resolution of ERP measures, it is difficult to determine the precise brain regions that impact the component measured on the scalp. Source localization techniques indicate that the striatum is a likely neural generator of the RewP (Foti et al., 2011). Further, studies examining the monetary reward domain using simultaneous EEG and fMRI (Becker et al., 2014) and asynchronous EEG and fMRI using the same task (Carlson et al., 2011; Foti et al., 2014) have linked the RewP to ventral striatal and vmPFC activation, and other studies suggest that social reward processing also engages these brain regions (Daniel & Pollmann, 2014; Izuma et al., 2008; Lin et al., 2012). At the same time, some studies have found unique neural substrates involved in social versus monetary reward feedback processing, such that social reward feedback also activates frontal and dorsal limbic regions (Chan et al., 2016; Rademacher et al., 2010).

Increasing evidence suggests that a blunted social RewP, relative to monetary RewP, may be uniquely associated with depressive symptoms and risk, given that it more closely indexes processes that impact responses to social stressors and interactions (Freeman et al., 2022; Hill et al., 2023; Kujawa, 2024; Oumeziane et al., 2019; Pegg et al., 2019, 2021). It is also important to note potential negative experiences associated with social media use, such as receiving negative peer feedback and the potential for increased rumination or negative social comparison (Feinstein et al., 2013; Koutamanis et al., 2015; Ohannessian et al., 2021), which may be occurring in conjunction with the receipt of social rewards. Considering other work demonstrating that high social reward sensitivity can attenuate the effect of psychosocial risk factors on depressive symptoms (Pegg et al., 2019, 2021), it could be that heightened social reward responsiveness protects against the harmful effects of these negative social media experiences on emotional health.

Taken together, extant literature suggests that social reward responsiveness may be a key process that moderates associations between adolescent social media use and affective health, but this has not yet been empirically tested. In the present study, we address this gap by examining the moderating role of social reward responsiveness (as measured by the RewP to peer acceptance feedback) on the association between social media use and momentary affect in adolescents at varying risk for depression. Further, we examined the specificity of *social* reward processing as a moderator by additionally testing the moderating effects of the RewP to monetary reward feedback on the relation between social media use and PA and NA. In our previous work with this sample, we found evidence that social media use reduces *both* PA and NA, though there was substantial heterogeneity in the effect of social media use on NA (Politte-Corn et al., 2024). Drawing from these findings and studies documenting protective effects of heightened social reward responsiveness (Pegg et al., 2019, 2021), we expected that heightened neural reactivity to social reward (i.e., peer acceptance) would protect against the harmful effects of social media use on reduced PA and/or higher NA. Additionally, we expected that this moderation effect would be specific to social versus monetary reward responsiveness.

## Method

### Participants

Participants were 156 adolescents aged 14–17 (*M* = 15.23; *SD* = 1.08) recruited as part of a study on social and emotional processing in depression risk and were oversampled for current clinical depression or risk for depression based on maternal history. Eleven participants either did not complete any EMA surveys (*n* = 9) or completed surveys that did not pass our data quality checks described below (*n* = 2) and were not included in the current analyses, resulting in a final sample of 145 participants. Of these, 50 were considered relatively low risk for depression based on no personal or maternal history of clinical depression, 47 had no history of clinical depression but were considered to be at high risk based on a maternal depression history, and 48 met diagnostic criteria for a current depressive disorder. Of these currently depressed youth, 16.3% met criteria for both major depressive disorder (MDD) and persistent depressive disorder (PDD; i.e., chronic MDD lastly at least 12 months), 32.7% met for MDD only, 20.4% met for PDD with intermittent major depressive episodes (MDE) including a current MDE, 22.4% met for PDD with intermittent MDE without current MDE, 6.1% met for PDD without a history of MDE, and one adolescent met for unspecified depression (defined as an MDE just short of the 2-week minimum or 5 symptom requirement with clinically significant distress or impairment). Of the final EMA sample, 140 participants also completed the EEG assessment. Across the two EEG tasks included in the current study, some data were excluded due to noisy data overall or at the mastoid electrodes used for data referencing (*n* = 8), data collection errors (*n* = 2), or because the participant wanted to exclude their data following the debrief (*n* = 1). Maximum likelihood estimation was used to account for missing data.

Regarding gender distribution, 90 participants (62.1%) identified as female, 53 (36.6%) identified as male, and 2 (1.4%) identified as nonbinary. As such, we were underpowered to examine associations between the study variables and each gender group and focus subsequent analyses on biological sex (female *n* = 93; male *n* = 52). The majority of participants identified as White (*n* = 104; 71.7%), 19 (13.1%) identified as Black or African American, 10 (6.9%) identified as Asian, 1 (0.7%) identified as American Indian or Alaska Native, 2 (1.4%) identified as Native Hawaiian or Pacific Islander, and 9 (6.2%) identified as another race. Nine participants (6.2%) further identified as Hispanic or Latinx. To assess socioeconomic status (SES), mothers reported on education level and annual income for themselves and a second parent, if applicable. Of the highest education level across both parents, 3 (2.1%) were high school graduates, 9 (6.2%) attended college or trade school but did not finish, 14 (9.7%) graduated a 2-year college or trade school, 45 (31.0%) graduated a 4-year college, 46 (31.7%) earned a Master’s degree, 26 (17.9%) earned a doctoral degree (e.g., MD, PhD, JD), and 2 (1.4%) did not report on education level. Of combined annual income across both parents, five (3.4%) reported income below $30,000, 37 (25.5%) between $30,000-$89,999, 53 (36.6%) between $90,000-$149,999, 28 (19.3%) between $150,000-$199,999, 20 (13.8%) reported an annual income of $200,000 or higher, and 2 (1.4%) did not report on annual income.

### Measures

#### EEG Tasks

##### Social Reward Task

Participants (*n* = 131) completed the Island Getaway task (Kujawa et al., 2014), which simulates social interactions between peers to elicit neural responses to social feedback. Previous studies have shown that ERPs sensitive to social reward (i.e., peer acceptance feedback) can be reliably measured in this task (Ethridge & Weinberg, 2018; Pegg et al., 2022). The task code is available at: https://github.com/Kodiologist/Survivor (branch: vanderbilt). In this task, participants were instructed that they would be playing a game with 11 other adolescents participating in studies in other labs around the U.S. In reality, all peers and responses were computerized. The participant’s goal was to be one of the 6 players who arrive at the “Big Island of Hawaii” without being kicked off by their peers. Prior to beginning the task, participants provided a photograph of themselves that was used to create their player profile. Next, participants answered several questions about their name, age, hometown, and general interests and reviewed the profiles of their peers.

In each round, participants were presented with the profile information of the other players and voted to either “keep” (accept) or “kick out” (reject) each peer, while led to believe that that peer was simultaneously voting to accept or reject them. To make the task more realistic, a statement appeared on the screen saying, “Waiting for [name] to vote…,” if participants voted faster than the simulated voting time assigned based on pilot testing. Following the vote, a fixation cross was presented for 1,000 ms, followed by peer feedback displayed for 2,000 ms. A green thumbs-up was shown on the screen indicating social reward/acceptance feedback, and a red thumbs-down was presented indicating social rejection. Participants then saw a blank screen for 1,500 ms before the next peer profile within the round was presented. At the end of each round, participants were shown the picture of the peer who was voted off during that round. All participants reached the final island at the end of the sixth and final round and were debriefed upon completion of the task. Over the course of 51 trials across six rounds, participants were presented with roughly equal acceptance and rejection feedback, but ultimately “won” the game without being voted out by peers. Prior work with young adults has shown that, on average, participants rate the task as moderately believable (Pegg et al., 2019).

##### Monetary Reward Task

Participants (*n* = 134) also completed the Doors reward task to measure monetary reward responsiveness (Bress et al., 2015; Kujawa et al., 2019). In this task, participants were presented with a pair of identical doors and instructed to guess which door had a prize behind it. All participants were informed that they could win up to $5 by guessing correctly across the task. After selecting a door, a fixation cross appeared for 1,000 ms, followed by feedback indicating whether they chose correctly for 1,500 ms. Two types of feedback were presented: a green up arrow indicating that they chose correctly and won $0.50 (monetary reward), or a red down arrow indicating that they chose incorrectly and lost $0.25. After feedback was presented, a fixation cross appeared for 1,000 ms and participants were then prompted to click for another round. The task included 30 win and 30 loss trials presented in a random order. All participants won the full $5 at the end of the task. The Doors task is widely used and can reliably elicit neural response to monetary reward across development (Ethridge et al., 2017; Pegg et al., 2020; Proudfit, 2015).

#### Ecological Momentary Assessment

Measures of social media use, PA and NA were obtained through EMA. Participants received text messages through SurveySignal seven times per day for one week (SurveySignal; Hofmann & Patel, 2015). The EMA period could start on any day of the week and at any time of year. Surveys were sent on a variable schedule and participants were given two hours to respond.^1^ Each survey prompted participants to report what they were doing at the moment including using social media, who they were with both in person and online, and current PA and NA. The mean number of assessments completed was 27.30 (*SD* = 12.72); in total, we obtained 4,052 assessments nested within 147 participants. Sixteen surveys were completed outside of the EMA period and were not included in the analyses. Further, to ascertain the quality of the EMA data, we excluded surveys completed in under 30 s (*n* = 39) or over 15 min (*n* = 63) and surveys completed within 15 min of the prior assessment (*n* = 24), resulting in a final sample of 3,910 assessments nested within 145 participants.

To assess whether EMA compliance was impacted by survey prompts sent during the school day, we examined potential differences in survey completion rates on weekdays versus weekend days and during the school year versus summer months. A paired samples t-test indicated that there were no significant differences in survey completion on weekend days (i.e., Saturday and Sunday) versus weekdays, *t*(144) = -1.55, *p* = .12, Cohen’s *d* = -0.002. Further, the average number of surveys completed did not significantly differ for participants who started the EMA period during the school year (i.e., between the months of August and May) compared to individuals who began the EMA period in June or July, *t*(144) = 1.64, *p* = 0.11, Cohen’s *d* = 0.07.

##### Social Media Use

On each survey, participants were asked “*What were you doing when you received this survey? Please select all that apply*” and presented with a checklist of several possible activities, the first of which was “*Browsing or posting on social media*.” As such, social media use was coded as either *0* (not browsing or posting on social media) or *1* (browsing or posting on social media) for each survey. Across all surveys included in the analyses, 463 (12.3%) included an endorsement of social media use. Of these observations, 337 (72.8%) were completed by female adolescents.

To more accurately characterize overall social media use in the current sample, we also asked participants to indicate the frequency of their social media use on a questionnaire. Specifically, participants were presented with several widely used social media platforms (based on estimates of the number of users and information from pilot testing) and rated how often they use each platform to connect or interact with other people (0 = *never,* 1 = *rarely,* 2 = *sometimes,* 3 = *pretty often,* 4 = *a lot*). Some participants (*n* = 91) also reported the average amount of time they spend on social media per day, as indicated by the screen time feature on their smartphones. This includes data from the past 7 or 10 days, depending on the smartphone’s operating system. Average time spent on common social media apps (namely, Facebook, Instagram, Snapchat, Twitter, and TikTok) ranged from 0 to 6.43 h per day, with an average of 1.26 h per day spent on these social media platforms.

##### Positive and Negative Affect

Affect was measured on each survey using a brief version of the Positive and Negative Affect Scales for Children (PANAS-C; Ebesutani et al., 2012; Laurent et al., 1999). Participants were asked to indicate the extent to which they felt each emotion at that moment. At each assessment, PA and NA were calculated by averaging responses to the 10 items which were rated on a Likert-type scale from 1 (very slightly or not at all) to 5 (extremely). PA was calculated by averaging five items (joyful, proud, cheerful, happy, and lively), and NA was measured by another five items (miserable, mad, scared, afraid, and sad). The PANAS scales display high internal consistency and strong convergent and discriminant validity with other measures of mood (Laurent et al., 1999) and the brief NA and PA scales display equivalent psychometric properties (Ebesutani et al., 2012). In the current sample, internal consistency was high for both PA (Cronbach’s alpha = .92) and NA (Cronbach’s alpha = .80).

### EEG Data Collection and Processing

EEG data were recorded using a 32-channel BrainProducts actiCHamp system and BrainVision Recorder software with a 1000 Hz sampling rate and impedances below 30 kΩ. Facial electrodes were attached approximately 1 cm above and below one eye and 1 cm from the outer corners of the eyes to measure electrooculogram (EOG) from eye movements, and referenced to an electrode placed on the back of the neck of the participant, per the BrainProducts bipolar-to-auxiliary adapter design. Early in the COVID-19 pandemic, a 16-channel system without facial electrodes was used for 20 participants to reduce the duration of contact with research staff (channels analyzed in the current study were available for all participants).^2^ EEG data were processed offline using BrainVision Analyzer software (Munich, Germany), filtered from 0.1 to 30 Hz, and re-referenced to an average of the two mastoids (TP9 and TP10). Data were segmented from 200 ms before to 800 ms after positive/negative feedback across both tasks. Data were corrected for eye movements (Gratton et al., 1983). For the subset of participants with the 16-electrode scheme, ocular correction was performed using FT9 in place of the horizontal facial electrodes with FT10 as the reference, and FP1 in place of the vertical facial electrodes with a common reference. Artifacts were removed using semi-automated procedures, with the following criteria for automatic artifact rejection: voltage step greater than 50 μV between sample points, maximum voltage difference of 175 μV within 400 ms intervals, minimal allowed amplitude of -200 μV and maximal allowed amplitude of 200 μV, and lowest allowed activity of 0.5 μV within 100 ms intervals. Additional artifacts were identified by visual inspection and removed. Following artifact rejection procedures, participants had on average 25.15 (*SD* = 1.00) trials for the accept condition, 25.19 (*SD* = 0.92) trials for the reject condition, 29.51 (*SD* = 1.69) for the win condition, and 29.57 (*SD* = 1.47) for the loss condition at Cz. Average ERPs were computed for each condition and baseline corrected to 200 ms preceding stimulus onset for both social and monetary feedback.

**Fig. 1:**
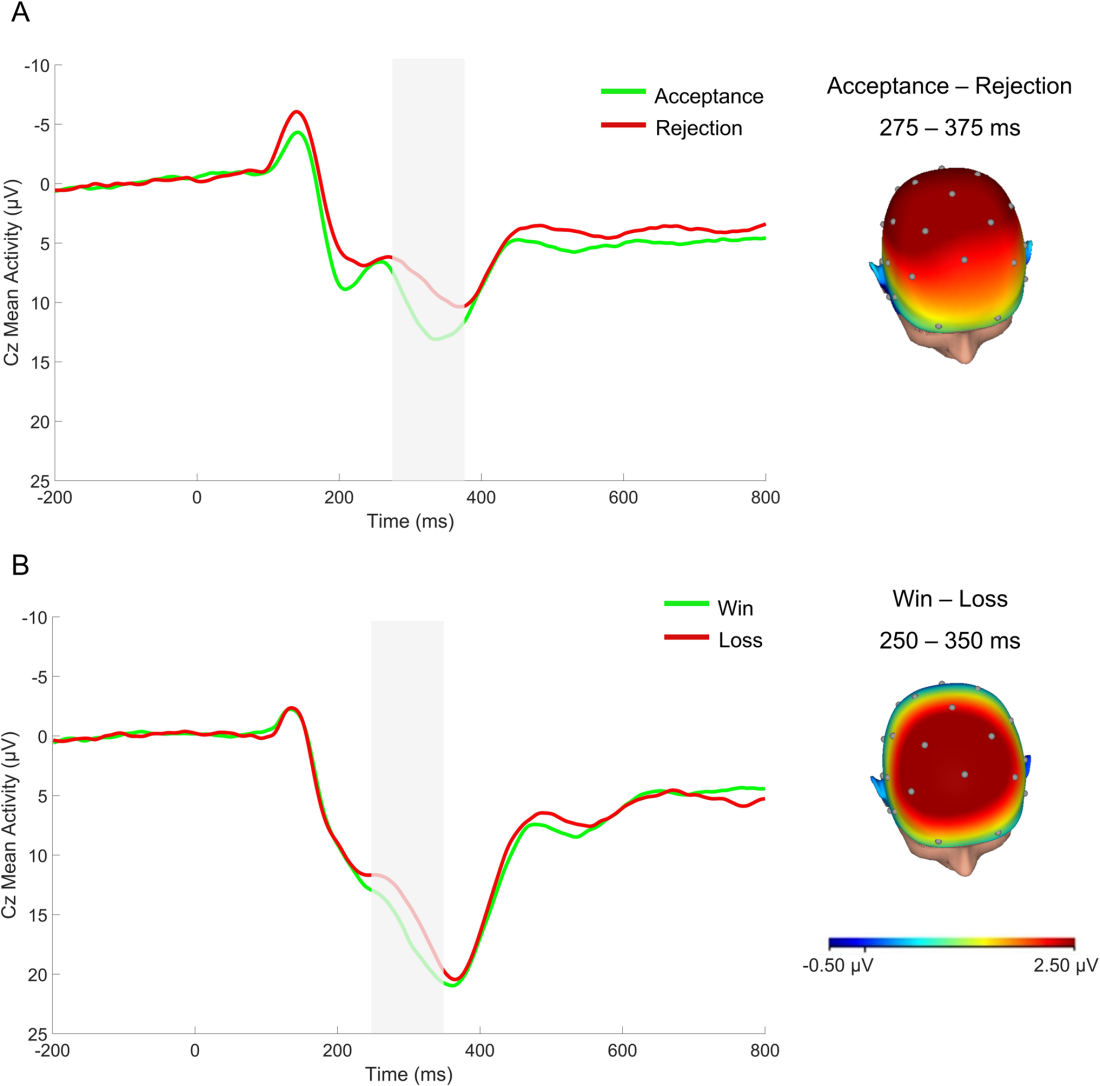
ERP waveforms (negative up) depicting responses to (**A**) social and (**B**) monetary feedback and scalp distributions depicting the response to reward vs. negative feedback in the time window for each RewP

Consistent with prior work and based on visual inspection of the grand averages in this study (Fig. 1), social RewP was extracted between 275-375 ms at Cz (Ethridge et al., 2017; Hill et al., 2023; Pegg et al., 2019; Rappaport et al., 2019) and monetary RewP was extracted between 250-350 ms at Cz (Foti & Hajcak, 2009; Pegg et al., 2020; Rappaport et al., 2019).^3^ We computed unstandardized residual RewP to acceptance/win, partialling out the variance associated with RewP to rejection/loss (Ethridge & Weinberg, 2018; Meyer et al., 2017). More positive values indicate greater neural response to social/monetary reward. RewP to acceptance and rejection feedback had high split-half reliability at Cz (Spearman-Brown coefficients = 0.90 and 0.88, respectively). RewP to win and loss feedback also demonstrated high split-half reliability at Cz (Spearman-Brown coefficients = 0.90 and 0.89, respectively).

### Data Analysis

We used multilevel modeling (also known as hierarchical linear modeling) to examine the moderating effects of neural measures of reward responsiveness (RewP) on the association between social media use and PA and NA. Multilevel modeling is also advantageous for analyzing EMA data because it does not assume that data points are independent and can handle missing values (Snijders & Bosker, 2011). The data were hierarchically arranged in three levels, with assessments (level 1) nested within days (level 2) nested within participants (level 3). Level-1 variables (measured at each assessment) included PA, NA, and social media use. We did not include any level-2 variables in the models. Level-3 variables (i.e., person-level variables) included residual RewP to social and monetary reward. We first imposed a lag-1 continuous autoregressive correlation structure on the residuals given time dependencies across assessments. Subsequently, we introduced cross-level interactions between social media use (using a fixed slope) and RewP residuals and probed significant interactions using simple slopes and the Johnson-Neyman region of significance. Given evidence that female adolescents are more sensitive to peer feedback than male adolescents (Guyer et al., 2012, 2014), we also conducted exploratory analyses to examine sex differences in the observed moderation effect. Finally, we examined exploratory three-way interactions between social media use, social/monetary RewP, and clinical group predicting concurrent PA and NA, given that depression is associated with alterations in PA and NA (Joiner & Lonigan, 2000; Lindahl & Archer, 2013), social functioning (Hammen, 2012), and neural reward responsiveness (Hill et al., 2023; Oumeziane et al., 2019; Pegg et al., 2019, 2021). Maximum likelihood estimation accounted for missing data (Peugh & Enders, 2004). All analyses were conducted in *R* using the *nlme* package (Pinheiro et al., 2020).

## Results

### Preliminary Analyses

ERP waveforms depicting social and monetary reward responsiveness effects and the corresponding scalp distributions are presented in Fig. 1. As expected, there were significant differences in average PA and NA across clinical groups [*F*(2,140) = 10.32, *p* < .001, η^2^ = 0.13 for PA; *F*(2,140) = 13.05, *p* < .001, η^2^ = 0.16 for NA], such that currently depressed adolescents reported lower average PA and higher average NA than the low- and high-risk groups (*p*s < .003). Average PA and NA did not significantly differ between the low- and high-risk groups (*p*s > .28). There were no significant group differences in frequency of social media use (the proportion of surveys in which social media use was endorsed^4^; *F*(2,135) = 1.65, *p* = .20, η^2^ = 0.02), social RewP [*F*(2,128) = 0.88, *p* = .42, η^2^ = 0.01], or monetary RewP [*F*(2,131) = 0.57, *p* = .57, η^2^ = 0.01]. Independent samples *t*-tests indicated that female adolescents reported lower average PA than male adolescents, *t*(141) = 3.08, *p* = .002, *d* = -0.56, but there were no significant sex differences in average NA, *t*(141) = -1.34, *p* = .18, *d* = 0.22, frequency of social media use, *t*(136) = -0.63, *p* = .53,* d* = 0.12, social RewP, *t*(129) = 1.70, *p* = .09, *d* = -0.31, or monetary RewP, *t*(132) = 1.53, *p* = .13, *d* = -0.27. Day of the week (i.e., weekday versus weekend) was not associated with affect, nor did the pattern of results change when including weekday as a level-2 covariate. As such, we report the results of the models without weekday entered as a covariate for parsimony.

Forty-two participants reported no social media use across surveys. Of these, 16 (38.1%) were in the low-risk group, 9 (21.4%) were in the high-risk group, and 17 (40.5%) were currently depressed. There was not a significant difference in the distribution of clinical groups between participants who did and did not report any social media use across EMA surveys [χ^2^(2) = 3.40, *p* = .18], nor did these participants differ from teens endorsing any social media use on average PA, NA, or social or monetary reward responsiveness (*p*s > .05). Further, there was not a significant difference between clinical groups in self-reported frequency of social media use [*F*(2,142) = 1.56, *p* = .21, η^2^ = 0.02]. However, clinical groups did significantly differ on objective screen time spent on social media [*F*(2,89) = 3.75, *p* = .03, η^2^ = 0.08], such that depressed adolescents spent more time on social media apps (*M* = 2.15 h per day) than the low-risk (*M* = 1.26 h per day) or high-risk (*M* = 1.22 h per day) groups (*ps* = .06). There was not a significant difference in social media screen time between low- and high-risk adolescents (*p* = .99).

Of those who reported no social media use across EMA surveys, only 10 (23.8%) indicated on the social media platform use questionnaire that they never use social media to connect or interact with other people. As such, most participants reporting no social media use on EMA surveys did have active social media accounts but were not using them at the time of the assessments. Thirty-one of these participants provided data on the average time they spent on social media apps per day, which ranged from 0 to 5.57 h (*M* = 0.52 h).

The ICCs for PA and NA supported the use of a three-level model, as there was a sizable amount of variance in affect that occurred across assessments, across days, and between subjects (37%, 8%, and 55% for PA; 37%, 10%, and 54% for NA). To examine associations between participants’ PA and NA, social media use, and social and monetary RewP, bivariate correlation analyses were first conducted (see Table 1). As expected, PA and NA were negatively correlated (between-person *r* = -.40; within-person *r* = -.32; *p*s < .001). Social media use was significantly correlated with PA at the within-person level (*r* = -.06, *p* < .001), but not at the between-person level (*r* = .00, *p* = .98). Monetary RewP residual scores were modestly correlated with average PA (*r* = .18). No other study variables were significantly correlated.

**Table 1:** Means, standard deviations, and correlations between the study variables

Variable	*M*	*SD*	1	2	3	4	5
1. Social media use	0.13	0.16					
2. Social RewP	0.00	5.33	.02				
3. Monetary RewP	0.00	4.64	-.08	.11			
4. PA	2.53	0.76	.00	.01	**.18**		
5. NA	1.31	0.41	-.07	.02	.07	**-.40**	
6. Number of assessments completed	27.30	12.72	-.15	.01	-.01	-.07	-.10

*Note*: Social media use reflects the proportion of surveys in which use of social media was endorsed; Social and monetary RewP represent unstandardized residual RewP to acceptance/win adjusting for RewP to rejection/loss, respectively; Statistically significant correlations at *p* < .05 are boldface

### Moderation Analyses

Four moderation analyses were conducted to examine relations between social media use, social and monetary RewP residual scores, and PA and NA (see Table 2). Specifically, we examined residual social and monetary RewP as moderators of the associations between social media use and PA and NA. We first entered social or monetary residual RewP as a level-3 predictor, covarying for concurrent social media use at level 1, to examine any conditional main effects. As seen in Table 2, social media use and a smaller monetary RewP were associated with lower PA (β = -0.05, *SE* = 0.01, *p* < .001 for social media use; β= 0.15, *SE* = 0.07, *p* = .03 for monetary RewP) There were no significant main effects on NA, nor did social RewP predict PA or NA when examined as a main effect.

**Table 2:** Regression analyses predicting concurrent momentary PA and NA from main and interactive effects of social media use, social RewP, and monetary RewP

**PA**		***β (SE)***	**t-value**	***p***			***β (SE)***	**t-value**	***p***
** Step 1**	Social media use	-.04 (.01)	-3.64	< .001	**Step 1**	Social media use	-.05 (.01)	-3.94	< .001
	Social RewP	.01 (.07)	0.12	.91		Monetary RewP	.15 (.07)	2.19	.03
** Step 2**	SMU X Social RewP	.03 (.01)	2.33	.02	**Step 2**	SMU X Monetary RewP	.003 (.01)	0.25	.81
**NA**		***β (SE)***	**t-value**	***p***			***β (SE)***	**t-value**	***p***
** Step 1**	Social media use	-.01 (.01)	-0.97	.33	**Step 1**	Social media use	-.02 (.02)	-0.80	.42
	Social RewP	.02 (.07)	0.30	.76		Monetary RewP	.01 (.01)	0.81	.42
** Step 2**	SMU X Social RewP	.003 (.02)	0.19	.85	**Step 2**	SMU X Monetary RewP	-.01 (.02)	-0.82	.41

Before adding cross-level interaction terms using social media use (level 1) and social or monetary RewP (level 3) to test moderating effects, we examined whether social media use should have a random slope. Likelihood ratio tests indicated that model fit was significantly improved upon inclusion of a random slope for social media use to predict NA (χ^2^(2) = 9.64, *p* = .047), but not PA (χ^2^(2) = 7.19, *p* = .13). Consequently, we used a fixed slope for social media use in subsequent models predicting PA and a random slope for social media use in models predicting NA.

**Fig. 2:**
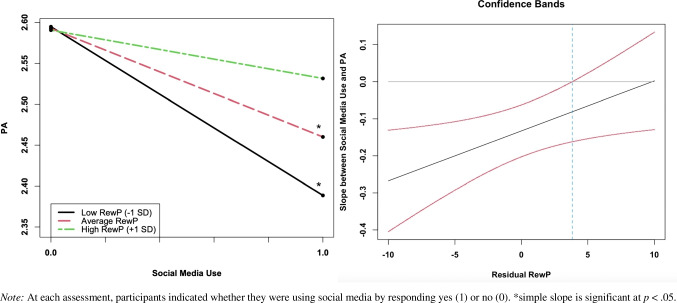
2-way interaction between social media use and social RewP predicting concurrent momentary PA in the full sample and Johnson-Neyman region of significance

As seen in Fig. 2, there was a significant interaction between social media use and social RewP predicting momentary PA [*β* = 0.03*, t*(128) = 2.33, *p* = .02]. Simple slopes analyses revealed that social media use was not significantly associated with concurrent PA for adolescents with a relatively enhanced (+ 1 *SD*) RewP to peer acceptance feedback (residual adjusting for responses to peer rejection) [simple slope = -0.06, *SE* = 0.05, *t* = -1.27, *p* = .20]. However, social media use was associated with lower concurrent PA for adolescents with low [simple slope = -0.21, *SE* = 0.05, *t* = -4.25, *p* < .001] or average [simple slope = -0.13, *SE* = 0.04, *t* = -3.74, *p* < .001] RewP to peer acceptance. The Johnson-Neyman region of significance indicated that the association between social media use and reduced PA was significant when residual social RewP was below 3.83 μV, to the left of the dashed line in Fig. 2. Further, this effect remained consistent when controlling for sex and when using a difference score for RewP rather than an unstandardized residual. There was no significant interaction between social media use and social RewP predicting NA, nor did monetary RewP significantly interact with social media use to predict momentary affect.

Because a cross-sectional association could indicate that adolescents with low social reward responsiveness and PA are more likely to use social media, we examined whether the observed interaction effect held when predicting later rather than concurrent PA and whether social RewP interacted with PA to predict social media use at the next assessment. When PA at the next assessment was entered as the outcome variable, the interaction between social media use and social RewP was no longer significant (*β* = 0.01, *SE* = 0.01, *p* = .41). Social RewP also did not interact with PA to predict lagged social media use (*β* = 0.001, *SE* = 0.002, *p* = .46).

### Exploratory Analyses

There was a significant three-way interaction between social media use, social RewP, and sex predicting concurrent PA [*β* = 0.13*,* *SE* = 0.06*, t*(126) = 1.99, *p* = .047]. Specifically, social RewP moderated the association between social media use and concurrent PA for female adolescents [*β* = 0.05*, z* = 1.99, *p* = .003], but not for male adolescents [*β* = *-*0.02*, z* = -1.08, *p* = .28]. The pattern of the interaction effect for female adolescents was consistent with that observed in the full sample, such that social media use was associated with lower PA only at average and low RewP to peer acceptance. There was not a significant three-way interaction between social media use, social RewP, and sex predicting concurrent NA [*β* = -0.09*,* *SE* = 0.07*, t*(126) = -1.25, *p* = .21]. We did not find significant differences in any of the interaction effects across clinical groups (*p*s > .12).

## Discussion

The present study was among the first to empirically examine the role of neural reactivity to social reward (i.e., peer acceptance feedback) in moderating the association between social media use and momentary affect in adolescents’ daily lives. Specifically, we found that among adolescents with blunted social reward responsiveness (i.e., a relatively reduced RewP to social acceptance feedback), social media use was associated with lower PA. However, social media use was not associated with PA for youth with enhanced social reward responsiveness. Further, although monetary RewP was related to PA overall, this moderation effect was specific to reward responsiveness in the social domain. These findings highlight heightened social reward sensitivity as a potential protective factor contributing to variability in the effect of social media use on emotional health.

Consistent with the study hypothesis, we found that social reward responsiveness moderated the effect of social media use on momentary PA. Specifically, heightened neural reactivity to peer acceptance attenuated the negative association between social media use and momentary PA. Exploratory analyses revealed that this moderation effect was specific to female adolescents. While social media use is characterized by various social rewards, such as likes and comments, prior studies have also highlighted deleterious social media experiences, such as co-rumination, negative peer feedback, and negative social comparison (Feinstein et al., 2013; Koutamanis et al., 2015; Ohannessian et al., 2021), all of which are particularly harmful for female adolescents (Nesi & Prinstein, 2015; Rose & Rudolph, 2006). As such, adolescents exhibiting heightened responsivity to social rewards may be less vulnerable to these negative experiences on social media, consistent with evidence of a stress-buffering effect of reward sensitivity (Goldstein et al., 2020; Pegg et al., 2019; Vidal-Ribas et al., 2019). Alternatively, reduced response to social reward could attenuate the beneficial effects of positive peer feedback on PA, consistent with a study indicating that low neural sensitivity to rewards reduced the beneficial effects of positive experiences on PA (Renault et al., 2023).

It is important to note that the moderation effect was only observed concurrently, indicating that social reward responsiveness does not appear to impact the effect of social media use on later PA. Further, social RewP did not interact with PA to predict subsequent social media use. This finding and the absence of a trait-level association between social RewP and social media use suggests that while neural sensitivity to rewards moderates the concurrent effect of social media use on PA, it does not seem to be associated with the frequency of or likelihood of engaging in social media use.

Notably, the moderating effect of social reward responsiveness on the association between social media use and affect was specific to PA rather than NA. In our previous work, we found evidence that social media use reduces *both* PA and NA, connoting both harmful and beneficial effects. The adolescent neurobiological sensitivity to context theory would suggest that adolescents with this high sensitivity to peer acceptance should be more susceptible to the benefits of social media usage (Schriber & Guyer, 2016). Drawing from this framework, we might expect a negative association between social media use and NA for adolescents with high social reward responsiveness. Instead, we found evidence that social reward responsiveness may protect against the harmful effects of social media on reduced PA, consistent with the stress-buffering hypothesis described above, rather than enhancing its beneficial effects on reduced NA. Given that reward responsiveness and PA are both conceptualized as components of positive valence systems (Insel et al., 2010; Kujawa et al., 2020; Olino, 2016), it could be that negative valence systems measures such as threat sensitivity are more relevant for characterizing social media effects on NA. However, specificity in the main effect of social media use and interactive effect of social media use and social reward responsiveness on PA highlights the role of positive valence system function in moderating associations between social media use and emotional health.

We also found specificity for social reward responsiveness as a moderator of the association between social media use and PA, as this effect did not emerge for monetary reward responsiveness. This is notable given that we used the same ERP component (RewP) to quantify reward responsiveness across both social and monetary domains. Further, this is consistent with recent conceptual models which posit that social media use engages areas of the brain implicated in social reward processing due to features such as likes and comments which connote quantifiable social rewards (Doheny & Lighthall, 2023; Meshi et al., 2015). It is also notable that we observed a main effect of monetary RewP, but not social RewP, on PA. This could suggest that neural response to monetary rewards reflects more general positive valence systems functioning, whereas social RewP may specifically reflect neurophysiological responses to social contexts. Other work has also found that reward responsiveness in the social domain, compared to monetary reward responsiveness, may be more relevant in moderating relations between social processes and affective outcomes (Hill et al., 2023; Pegg et al., 2021; Rappaport et al., 2019), perhaps due to developmental changes in social reward sensitivity and the role of interpersonal relationships in risk and maintenance of affective psychopathology (Blakemore & Mills, 2014; Hammen, 2005; Nelson et al., 2016).

Strengths of the current study include the use of EMA to delineate temporal associations between social media use, social reward responsiveness, and affect, the inclusion of multiple measures of reward responsiveness across social and monetary domains, and the use of hierarchical linear modeling to leverage all EMA data and minimize standard errors. However, there are also some limitations that could guide future work. First, our measure of social media use did not capture specific behaviors or experiences that may differentially interact with social reward sensitivity to predict affect. For example, other studies have assessed positive and negative experiences on social media and their unique associations with affective outcomes (Hamilton et al., 2021; Nesi et al., 2022). This approach could further delineate whether social reward responsiveness protects against negative social media experiences or rather enhances the beneficial effects of positive online interactions. Relatedly, our measure did not differentiate active versus passive use of social media, which may be an important factor influencing affect. We also did not define “social media” as part of our measure, so it is possible that participants misinterpreted what should be counted as such. Second, we observed substantial heterogeneity in the effect of social media use on NA in the current sample (Politte-Corn et al., 2024), but this effect was not moderated by reward responsiveness. As such, elucidating between-person variability in social media effects on NA remains an important direction for future work. Finally, our sample was predominantly White and high SES. As in the offline world, adolescents of color are also exposed to racial discrimination online (Tao & Fisher, 2022; Thomas et al., 2023) and social media has emerged as an avenue for identity exploration and development among minoritized youth (Brinkman & Jacobi, 2020; LeBlanc & Loyd, 2022). Moreover, trajectories of depressive symptoms across adolescence differ across racial and ethnic groups (Brown et al., 2007; Sen, 2004). Consequently, ethnic and racial minority youth may have unique experiences on social media that differentially relate to social reward responsiveness and affective outcomes.

The current study examined the moderating role of neural response to social reward (i.e., peer acceptance) on the association between social media use and momentary affect. Consistent with our hypothesis, heightened reward responsiveness attenuated the association between social media use and low PA, and this effect was specific to adolescent females and to social versus monetary reward. However, social reward responsiveness did not moderate the effect of social media use on NA. Results highlight individual differences in social reward sensitivity as a potential protective factor contributing to variability in the effect of social media use on emotional health in adolescents. Future studies can build on these findings by examining specific social media experiences and elucidating variability in social media effects on NA.

## Footnotes

1 Fifty participants received surveys between 6:25am and 10 pm. Due to a change in software, 95 participants were set on a different time zone and received surveys between 8:25am-12am, and we covaried for this time difference in analyses.

2 The pattern of results did not change when covarying for the number of channels used during EEG data collection.

3 The pattern of results did not change when using the 250–350 ms time window for social RewP.

4 Participants who completed < 5 assessments (*n* = 7) were not included in analyses with this variable.

## Additional Information

### Data Availability

Data are available upon reasonable request to A. Kujawa (autumn.kujawa@vanderbilt.edu).

### Code Availability

Code is available upon request from the corresponding author. This study was not pre-registered.
